# Multiple roles for Bcl-3 in mammary gland branching, stromal collagen invasion, involution and tumor pathology

**DOI:** 10.1186/s13058-022-01536-w

**Published:** 2022-06-09

**Authors:** David Carr, Aiman Zein, Josée Coulombe, Tianqi Jiang, Miguel A. Cabrita, Gwendoline Ward, Manijeh Daneshmand, Andrea Sau, M. A. Christine Pratt

**Affiliations:** Department of Cellular and Molecular Medicine, University of Ottawa, 451 Smyth Road, Ottawa, ON K1H 8M5 Canada

**Keywords:** Bcl-3, Branching morphogenesis, Extracellular matrix invasion, Post-lactational involution, Bcl-2, Apoptosis, Tumor pathology

## Abstract

**Background:**

The Bcl-3 protein is an atypical member of the inhibitor of -κB family that has dual roles as a transcriptional repressor and a coactivator for dimers of NF-κB p50 and p52. Bcl-3 is expressed in mammary adenocarcinomas and can promote tumorigenesis and survival signaling and has a key role in tumor metastasis. In this study, we have investigated the role of Bcl-3 in the normal mammary gland and impact on tumor pathology.

**Methods:**

We utilized *bcl-3*^*−/−*^ mice to study mammary gland structure in virgins and during gestation, lactation and early involution. Expression of involution-associated genes and proteins and putative Bcl-3 target genes was examined by qRT-PCR and immunoblot analysis. Cell autonomous branching morphogenesis and collagen I invasion properties of *bcl-3*^*−/−*^ organoids were tested in 3D hydrogel cultures. The role of Bcl-3 in tumorigenesis and tumor pathology was also assessed using a stochastic carcinogen-induced mammary tumor model.

**Results:**

*Bcl-3*^*−/−*^ mammary glands demonstrated reduced branching complexity in virgin and pregnant mice. This defect was recapitulated in vitro where significant defects in bud formation were observed in *bcl-3*^*−/−*^ mammary organoid cultures. *Bcl-3*^*−/−*^ organoids showed a striking defect in protrusive collective fibrillary collagen I invasion associated with reduced expression of Fzd1 and Twist2. Virgin and pregnant *bcl-3*^*−/−*^ glands showed increased apoptosis and rapid increases in lysosomal cell death and apoptosis after forced weaning compared to WT mice. Bcl-2 and Id3 are strongly induced in WT but not *bcl-3*^*−/−*^ glands in early involution. Tumors in WT mice were predominately adenocarcinomas with NF-κB activation, while *bcl-3*^*−/−*^ lesions were largely squamous lacking NF-κB and with low Bcl-2 expression.

**Conclusions:**

Collectively, our results demonstrate that Bcl-3 has a key function in mammary gland branching morphogenesis, in part by regulation of genes involved in extracellular matrix invasion. Markedly reduced levels of pro-survival proteins expression in *bcl-3* null compared to WT glands 24 h post-weaning indicate that Bcl-3 has a role in moderating the rate of early phase involution. Lastly, a reduced incidence of *bcl-3*^*−/−*^ mammary adenocarcinomas versus squamous lesions indicates that Bcl-3 supports the progression of epithelial but not metaplastic cancers.

**Supplementary Information:**

The online version contains supplementary material available at 10.1186/s13058-022-01536-w.

## Background

NF-κB signaling has a major role in normal mammary gland development where it contributes to both proliferation as well as cell death [1, 2]. The NF-κB family of transcription factors consists of p105 (NF-κB1) and p100 (NF-κB2), p65 (RelA) and RelB. p100 and p105 undergo inducible and constitutive partial proteolytic processing, respectively, resulting in p50 and p52 which preferentially form heterodimers with p65 and RelB proteins. p65 and p50 are constituents of the canonical NF-κB pathway and are retained in the cytoplasm through interaction with the inhibitor of -κB protein, IκBα. Phosphorylation of IκBα mediated by the IκB kinase complex (IKKα/IKKβ) through stimulation of receptors in the tumor necrosis family and other intracellular signaling pathways results in proteasome-mediated IκBα degradation allowing nuclear translocation of p65/p50. The alternative NF-κB pathway is stimulated by the NF-κB-inducing kinase (NIK) which induces IKKα homodimer-mediated p100 phosphorylation to signal partial processing to p52 and the ensuing nuclear translocation of p52/RelB heterodimers [2, 3]. Both p52 and p50 lack transactivation domains [3]; however, p50 and p52 homodimers can also interact with the NF-κB/IκB family protein, Bcl-3, resulting in either transcriptional activation or inhibition [4–7]. The *Bcl-3* gene was first identified at the breakpoint in the t(14;19) translocation in chronic lymphocytic leukemia in which it is overexpressed. Bcl-3 transgenic mice develop B cell accumulations at various sites, while Bcl-3 null mice have severely impaired humoral immune response [8, 9]. Bcl-3 expression and function have been associated with the promotion of stemness in both normal [10, 11] and cancer cells [12, 13].

Bcl-3 is activated downstream of signal transducer and activator of transcription 3 (Stat3) signaling [10] which in turn plays a critical role in growth and cell death in the mammary gland [14]. Moreover, Bcl-3 can induce the STAT3 gene in human cancer cells [15], while STAT3 can reciprocally induce Bcl-3 expression in mammary epithelial cells [16].

Bcl-3 also has pro-survival functions exemplified by the observation that Bcl-3/p52 complexes can induce the anti-apoptotic protein, Bcl-2 [17]. An important negative regulatory loop was also shown to exist between Bcl-3 and p53 wherein they are mutually repressive. The p53 protein induces a switch from the Bcl-3/p52 transcriptional activator complex to a Bcl-3/HDAC1 repressor complex [18], while Bcl-3 can reduce p53 stability in part through increasing levels of Hdm2 [19]. Therefore, Bcl-3 is strongly implicated in survival signaling.

The mammary gland is composed of a basal and luminal epithelium arranged as ducts and alveolar terminal ductal units that derive from mammary stem/bipotent cells and a spectrum of differentiating luminal progenitor cell populations [20]. Ductal branching morphogenesis is initiated in the embryo comprising a primary duct and a few secondary branches in humans. At puberty, hormones and growth factors induce further secondary and tertiary branching and elongation of these structures throughout the mammary fat pad and stromal connective tissue [21]. Fibrous collagenous connective tissue constitutes a large proportion of the stroma particularly in the human mammary gland [22].

In the adult, cells in the mammary gland undergo successive rounds of proliferation and cell death during pregnancy and involution [23]. Pregnancy elicits further secondary and tertiary branching in the mammary gland as progesterone induces signaling that stimulates progenitor cells to proliferate and generating the lobuloalveolar units that terminally differentiate near parturition to produce milk [24–26]. At weaning, the gland undergoes the process of involution that takes place in two phases [27]. The first phase is initiated by milk engorgement which stimulates production of leukemia inhibitory factor (LIF) [28, 29] which then activates STAT3. STAT3 signaling initiates and regulates the involution transcriptional program. Involution is reversible within the first 2 days after weaning in mice, implicating cell survival mechanisms during this period, which gradually progresses after 48 h toward the second phase involving extensive apoptosis combined with macroautophagy of the alveolar epithelial cells [30] followed by 6–8 days of irreversible structural mammary gland remodeling [27, 31].

Bcl-3 has been reported to be instrumental in promoting mammary tumor metastasis in two mouse models of breast cancer [32, 33]; however, the role of Bcl-3 in normal mammary gland development and function has not been characterized. Here, we show that mice null for Bcl-3 demonstrate reduced secondary mammary branching and organoids display a marked deficit in ability to form budding structures in basement membrane cultures and invade 3D collagen I. Absence of Bcl-3 also resulted in increased mammary gland apoptosis and accelerated post-lactational involution associated with perturbed regulation of the Bcl-3 target gene, Bcl-2. The proportion of carcinogen-induced mammary tumors arising in *bcl-3*^*−/−*^ mice was strongly biased toward a smooth muscle actin negative squamous phenotype characterized by low Bcl-2 expression and absence of NF-κB activity. Overall these results demonstrate that Bcl-3 is an important regulator of normal mammary gland biology and contributes to the development and/or progression of mammary adenocarcinoma, at least in part, as a consequence Bcl-2 regulation.

## Materials and methods

### Mice

C57/Bl6 *bcl-3*^*−/−*^ mice [8] have been previously described and were bred in homozygous matings, and C57/Bl6 mice (Taconic) were used as controls or *bcl-3*^+*/*+^ littermates. Virgin female mice were killed at 8–10 weeks of age. Virgin mice were estrus phase-matched based on vaginal cell smears at 6 weeks of age. Pregnant and lactating mice were killed at different days after the appearance of a vaginal plug (days of pregnancy/d$x$P) or after pups were born (days of lactation; d$x$L) or after pups were removed (24 h post-weaning/involution, 24hI). Weaning took place on the morning of d21L or at d10L as indicated. Some dams were also killed on the morning of d19L. Male mice were removed from the cages one day after appearance of the vaginal plug. Litters were monitored for suckling intermittently. The fourth inguinal mammary glands were removed and used for analysis. For tumorigenesis experiments, we used FVB/n *bcl-3* null mice [34] (Jackson Labs) used previously in tumor model studies [32]. Genotyping of C57/bl6 WT and *bcl-3*^*−/−*^ ear punch DNA was performed using two reverse PCR primers: CAGGCTGTTGTTCTCCACG (WT) allele and CATACGCTTGATCCGGCTAC (NEO sequence/knockout) with a common forward primer GTGGCGCGGACATCGATG. Animals were housed on a protocol approved by the University of Ottawa Animal Care and Veterinary Services Committee and in accordance with Canadian Council on Animal Care guidelines.

### Mammary gland whole mounts and analysis

Fourth inguinal mammary glands were excised from mice at diestrus and spread onto glass microscope slides. Glands were fixed overnight in Carnoy’s fixative (10% glacial acetic acid, 30% chloroform, 60% ethanol). Fixed glands were washed in 70%, 50%, 25% ethanol and then distilled water for 15 min each, and then, fat was extracted in acetone following 3–20 min incubations. Glands were rehydrated in 100% ethanol and then 95% ethanol for 20 min each and then stained with hematoxylin for one hour at room temperature. After rinsing clear in water, specimens were de-stained by submerging in acid alcohol (50% ethanol, 0.2% HCl) twice for 30 min and then in fresh acid alcohol overnight. Finally, glands were dehydrated in 70%, 95 and 100% ethanol for 20 min each and then stored in toluene. Samples were mounted using Permount (Fisher). Branch points were enumerated on the same duct relative to the nipple and lymph node on mammary glands from 3 different mice per genotype. Whole glands were imaged on an EVOS FL Auto 2 microscope.

### Estrus staging

Cells were obtained after flushing the vaginal canal with 100ul of saline, and a drop of the sample was smeared on a microscope slide, allowed to air-dry and stained with 0.1% toluidine blue. Sample smears are shown in Additional file 1: Fig. S1.

### Immunoblots and antibodies

Total mammary gland and tumor protein extracts were obtained from snap frozen tissue that was pulverized under liquid N2 as described [35]. After the addition of RIPA buffer (50 mM Tris–HCl, 150 mM NaCl, 1% NP-40, 0.5% sodium deoxycholate, 0.1% SDS, pH 7.5, 10 μg/ml aprotinin, 1 μM pepstatin, 1 μM leupeptin, 1 mM phenylmethylsulfonyl fluoride (PMSF), 1 mM Na_2_VO_4)_, the samples were sonicated then incubated on ice for 30 min. Insoluble material was removed by centrifugation at 9000* g* for 20 min at 4 °C. Protein samples were aliquoted and frozen at − 80C. Densitometry was performed using ImageJ.

Antibodies: β-actin #A-2066; SMA #ABT1487 (both Sigma), P-Stat3 #9134, Stat3 #9139, p53(D2H90) #32532S, Bim #2433S (all from Cell Signaling), Puma #ab54288, vinculin #ab129003, Smad3 #ab84177, (all Abcam); gapdh (Biolegend, #MMS-5805); NF-κB2(p52) (#06–413), Bcl-2 (#5826) (both Upstate); RelB (C-19) #sc-226, p65/RelA #sc-109, p50(H-119) #sc7178, Id3(2B11) #sc-56712, (all Santa Cruz).

### Mammary gland histology

Mammary glands were removed immediately after euthanization of mice, and the fourth inguinal gland was placed in 10% formalin. Mouse tissues were paraffin-embedded, and sections were prepared and stained with hematoxylin and eosin (H&E) using standard procedures by the University of Ottawa Department of Pathology and Laboratory Medicine. Briefly, slides were deparaffinized in toluene, hydrated (100%, 95%, 70% ethanol and water, 10 min each). Dehydration was performed in 70%, 95% and 100% ethanol followed by toluene. H&E-stained sections (5 µm) were mounted with coverslips using permanent mounting media (Permount, ThermoFisher). Sections were visualized and imaged using a Zeiss Axiophot fluorescence microscope equipped with Northern Eclipse software (EMPIX Imaging Inc., Mississauga, ON). Epithelial/adipose areas were circumscribed and calculated in Adobe Photoshop.

### 3D basement membrane and collagen I organoid cultures

Mammary epithelial organoids were collected from fourth inguinal mammary gland of 8–12-week-old virgin mice, and finely minced tissue was transferred to a digestion solution consisting of serum-free D-MEM/F-12 (Gibco) and 2 mg ml^−1^ collagenase A (StemCell Technologies). This was incubated for 1.5–2 h at 37C with rotation to liberate epithelial tissue fragments (‘organoids’). Isolated organoids were treated with DNAse I at 2 U/ml for 3 min at RT and centrifuged at 400 × g for 5 min. The organoids were resuspended in 5% calf serum in PBS and were subjected to a series of brief centrifugation to eliminate single cells. The purified organoids were resuspended at a density of 2 organoids/ul in Cultrex 3D BME or Cultrex Collagen I (Trevigen), and 50 μl were seeded per well of 24-well plates. The culture medium contained D-MEM/F-12 with penicillin/streptomycin, 10 mM HEPES, Glutamax and B27 supplemented with EGF (50 ng ml^−1^, Sigma Aldrich). 500 μl of culture medium was added per well, and organoids were maintained in a 37C humidified atmosphere under 5% CO_2_. Organoid phase contrast images were acquired using an EVOS FL Auto 2 microscope (Invitrogen). To collect cells from collagen I hydrogels, organoid domes were washed twice with PBS and then incubated for 90 min in 500ul Cultrex Organoid Harvesting Solution (R&D systems #3700-100-01) at 4C with slow rocking. The organoids then were treated with 2 mg/ml Collagenase Type I in PBS for 15 min and then incubated in 0.25% trypsin at 37C and 5% CO_2_ to digest the organoids into single cells. Cell viability was assessed by mixing 10 ul of 0.4% trypan blue with 10ul of each cell suspension and counted using a Countess II FL automated cell counter (Invitrogen), H&E-stained.

### Histochemistry, immunohistochemistry and immunofluorescence

Formalin-fixed, paraffin-embedded tissue sections were used for hematoxylin and eosin staining and for analysis of apoptotic cells using the Apoptag kit (EMD Millipore) for TUNEL labeling according to the manufacturer’s directions. Tumor tissue was also frozen in optimal cutting temperature compound (OCT) (Sigma). Cryostat sections of 5–7 μm were placed on Superfrost/Plus glass slides (Fisher Scientific, ON). Sections were fixed in either 4% paraformaldehyde or cold 100% methanol. Slides were washed in phosphate-buffered saline (3 × 5 min) , and then, blocking solution (0.2 mM Tris HCl, 3% Triton X-100, 1% BSA, 1% normal goat serum) was applied to cover the sections for 1 h at RT. Primary antibodies were incubated for 3 h followed by 3 × 5 min PBS washes. Cy3 conjugate (Jackson Labs) was used to detect immunoreactivity. Slides were counterstained with DAPI and mounted with Prolong anti-fade medium (Invitrogen).

Organoids in Cultrex BME were fixed with 4% (wt/vol) paraformaldehyde for 20 min, rinsed twice in PBS for 10 min and incubated in 30% sucrose for 72 h at 4C and then 30% sucrose/OCT mixture (1:1) for 4 h at 4C. Samples were then embedded in OCT and frozen at − 80 °C. 10-μm cryostat sections were obtained at − 25C. Samples on slides were washed in PBS (3 × 5 min), permeabilized with 0.3% (vol/vol) Triton X-100 in PBS for 20 min and then incubated in blocking solution (0.05% (vol/vol) Triton X-100 PBS and 5% goat serum). The sections were then incubated in blocking solution with anti-Bcl-3 (Invitrogen 23959-1-AP) overnight in 1:100 dilution. Sections were then washed by PBS (3 × 5 min) before incubation in anti-rabbit HRP for 1 h at RT. The sections were washed by PBS (3 × 5 min) and mounted in DAPI-containing Prolong anti-fade medium. Fluorescent imaging was performed using a Zeiss Axiophot D1 fluorescence microscope equipped with Axiovision software.

### Electrophoretic mobility shift assays (EMSA)

Nuclear extracts of tumors and EMSA analysis were performed essentially as we have previously described [35] modified from Osborn et al. [36]. NF-κB site oligonucleotides were obtained from Promega (E3291) and end labeled with T4 polynucleotide kinase by using [γ-^32^P]ATP (Amersham). Five micrograms of nuclear extract was mixed with 5 μl of DNA binding buffer (20 mM HEPES [pH 7.9], 0.2 mM EDTA, 0.2 mM EGTA and 2 mM dithiothreitol in 50% glycerol), 5 μg of poly(dI-dC) and 0.2 ng of labeled probe in a final volume of 20 μl and then incubated at room temperature for 25 min. Samples were loaded on a 5% native polyacrylamide gel and run in non-denaturing Tris–glycine buffer. A 50-fold excess of unlabeled NF-κB probe was used as a control for binding. For supershift analysis, 2ug of antibody or non-immune goat serum was added on ice for 10 min prior to gel loading.

### DMBA-MPA tumor model

For mammary tumor formation, 30 control and 30 *bcl-3*^*−/−*^ females were used. All animals were implanted with a medroxy-progesterone release pellet (Innovative Research, Hialeah, FL) at 6 weeks of age. Mice were gavaged at 9 and 10 weeks and again at 12 and 13 weeks of age with 50 mg/kg DMBA in cottonseed oil as described [37]. Animals were monitored for tumor formation three times weekly by palpation and tumors removed when they reached 1cm^3^ endpoint. The proportion of squamous and microacinar pathologies in tumors were estimated within circumscribed areas using Adobe Photoshop.

### qRT-PCR

Mammary glands were stored in RNAlater (ThermoFisher Scientific). Total RNA was isolated using Tripure Isolation Reagent (Roche) or Qiazol (Qiagen) according to the manufacturer’s protocol and was resuspended in 20 ul of nuclease-free water. cDNA was generated by reverse transcribing five μg of RNA with the RevertAid H Minus reverse transcriptase (Fermentas/ThermoFisher) or Superscript III (Invitrogen/ThermoFisher). The resulting cDNA was diluted 1:20 and used in a qRT-PCR reaction with FASTStart Universal SYBR Green Master Mix (Roche). All qRT-PCR reactions were performed with an ABI 7500 Real-Time PCR System (ABI/ThermoFisher), and relative gene expression was quantitated using the delta C_T_ method. Primers were designed online using the Roche Universal Probe Library Assay Design Center (https://lifescience.roche.com/shop/CategoryDisplay?catalogId=10001&tab=&identifier=Universal+Probe+Library) and manufactured by Invitrogen/ThermoFisher. Murine β-actin (*Actnb*) or 18S rRNA was used as the endogenous reference cDNA. Data are representative of 3 independent mice performed in triplicate.

### Statistics

Results were expressed as mean ± S.E.M. of three or more separate experiments. Student’s *t* test and one-way ANOVA analysis was done using GraphPad Prism software. A minimum *p* value < 0.05 was considered statistically significant.

## Results

### *Bcl-3* null mammary glands have reduced ductal branching

**Fig. 1:**
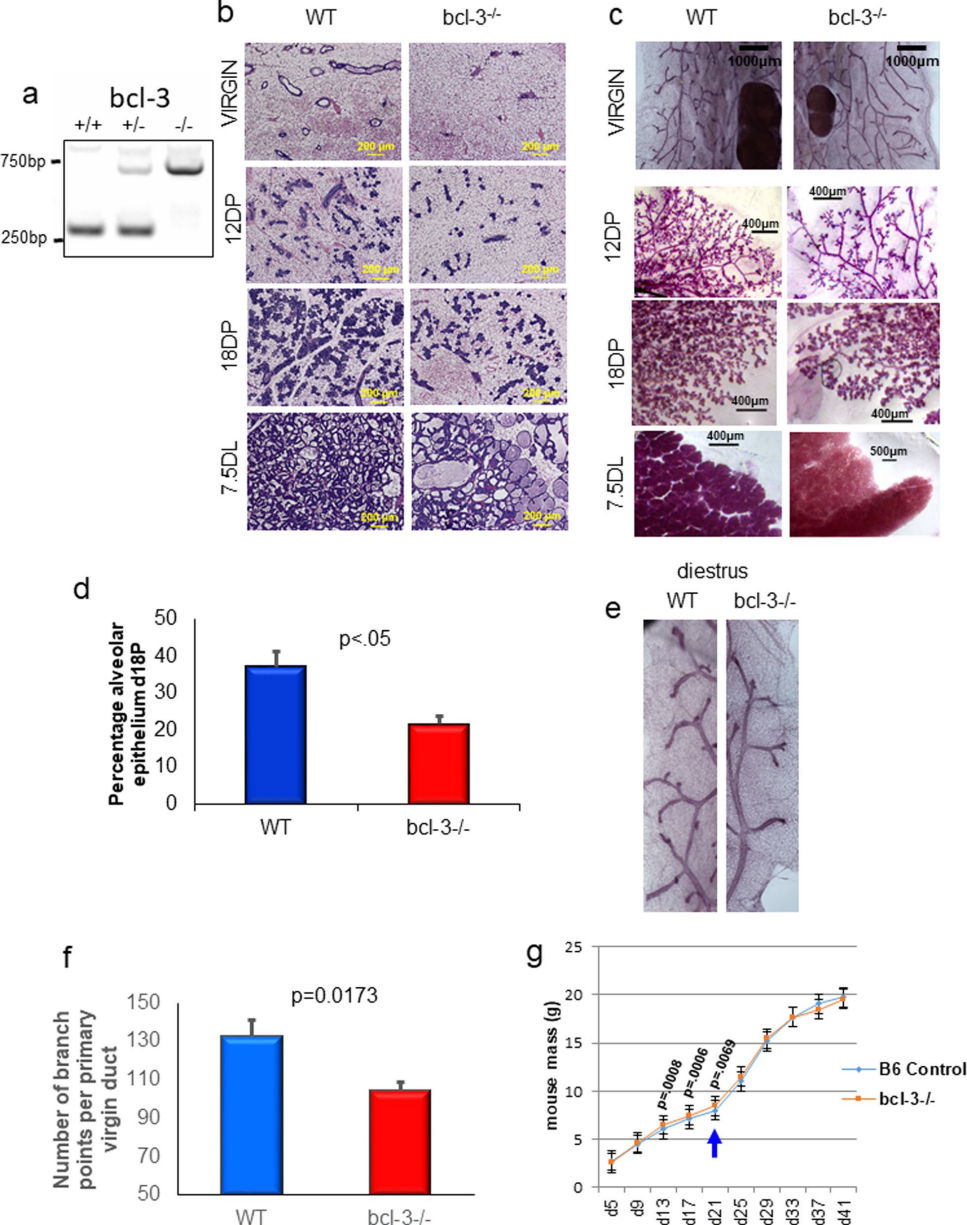
*Bcl-3*^*−/−*^ mice have reduced mammary branching but no lactation defect. **a** PCR analysis of *bcl-3* allele genotypes. +/+ and −/*− *mice were used for these studies. Figures represent results from 3 mice per stage. **b** WT and *bcl-3*^*−/−*^ mouse mammary glands were collected from 6-week-old virgin mice and from 12 and 18 days of pregnancy and day 7.5 of lactation. Sections of paraffin-embedded mammary glands were stained with H&E. **c** Representative whole mounts of WT and *bcl-3*^*−/−*^ mammary glands at the indicated stages. **d** The percentage of alveolar epithelium was calculated from H&E-stained glands at d18P. **e** Magnification of ductal branching from WT and *bcl-3*^*−/−*^ whole mounts at 6 weeks (diestrus). **f** Secondary and tertiary branch points were enumerated from diestrus virgin mouse gland whole mounts at 6 weeks. Bars are S.E.M. Statistical analysis; unpaired *t* test. **g** Pup masses were determined at various days of lactation and after weaning at day 21 after birth (blue arrow). Bars are mean mass of 32 WT and 18 *bcl-3*^*−/−*^ pups from 3 dams per genotype. Bars are S.D. *p* values indicate significantly greater mass of *bcl-3*^*−/−*^ pups compared to WT at the indicated days of lactation (unpaired *t* test). WT and *bcl-3*^*−/−*^ pup masses on all other days were not significantly different

Canonical and alternative NF-κB has important roles in the development of the mammary gland during pregnancy [2] and during involution [38]. Aberrant activation of the canonical pathway results in mammary epithelial proliferation and aberrant secondary ductal branching in the adult and during early postnatal development [39]. Enforced canonical signaling results in a delay in ductal branching while ectopic alternative NF-κB expression delays ductal development and impairs secondary branching during pregnancy [40]. Thus, regulation of NF-κB is critical for normal mammary gland development [41]. The specific role of Bcl-3 in mammary gland development at any stage has not been reported. We therefore examined the effect of the absence of Bcl-3 on mammary gland structure in virgin mice and at various stages of the pregnancy lactation cycle. *Bcl-3*^*−/−*^ and WT animals were confirmed by PCR genotyping analysis (Fig. 1a), and mammary glands were isolated from 8–10-week-old virgin mice in diestrus and at different stages of pregnancy, lactation and at day 1 of involution. Paraffin sections and whole mount images of glands in Fig. 1b, c, respectively, show reduced complexity of ductal bifurcation in virgin *bcl-3*^*−/−*^ mice. Side branching at day 12 of pregnancy (d12P) was also reduced. Alveologenesis appeared normal at day 18 of pregnancy (d18P), but alveoli were fewer in number compared to controls on a background of reduced branching in *bcl-3*^*−/−*^ mice. Quantification of alveolar areas indicated a significant decrease in the percentage of alveolar epithelium in *bcl-3*^*−/−*^ glands relative to WT glands at d18P (Fig. 1d). At day 7.5 of lactation (d7.5L), many *bcl-3*^*−/−*^ alveoli are much larger than WT alveoli. Close inspection of ductal networks (Fig. 1e) indicated reduced branching of *bcl-3*^*−/−*^ ducts compared to WT which was confirmed by quantification of branch points in 6-week-old virgin mice at diestrus (Fig. 1f). To determine if reduced complexity of *bcl-3*^*−/−*^ mammary glands affected pup growth, we weighed pups during the course of nursing and for several days after weaning at 21 days of lactation. *Bcl-3*^*−/−*^ pups had significantly greater mean mass at 13, 17 and 21 days old, but no other significant differences were obtained. Mean litter size was 11 in WT mice and 6 in *bcl-3*^*−/−*^ mice which may account for the larger average weight of pups in the last 2 weeks of suckling. Thus, given the difference in litter sizes, it is not possible to discern whether milk production in *bcl-3*^*−/−*^ mice was altered relative to WT mice (Fig. 1g). After weaning, no differences were observed.

Thus, virgin mammary development at the level of secondary branching is attenuated in *bcl-3*^*−/−*^ mice and evidence of hyper-enlarged alveoli is evident in mid-lactation that does not affect milk production.

### Absence of Bcl-3 limits mammary branching in vitro and is critical for collective cell collagen invasion

**Fig. 2:**
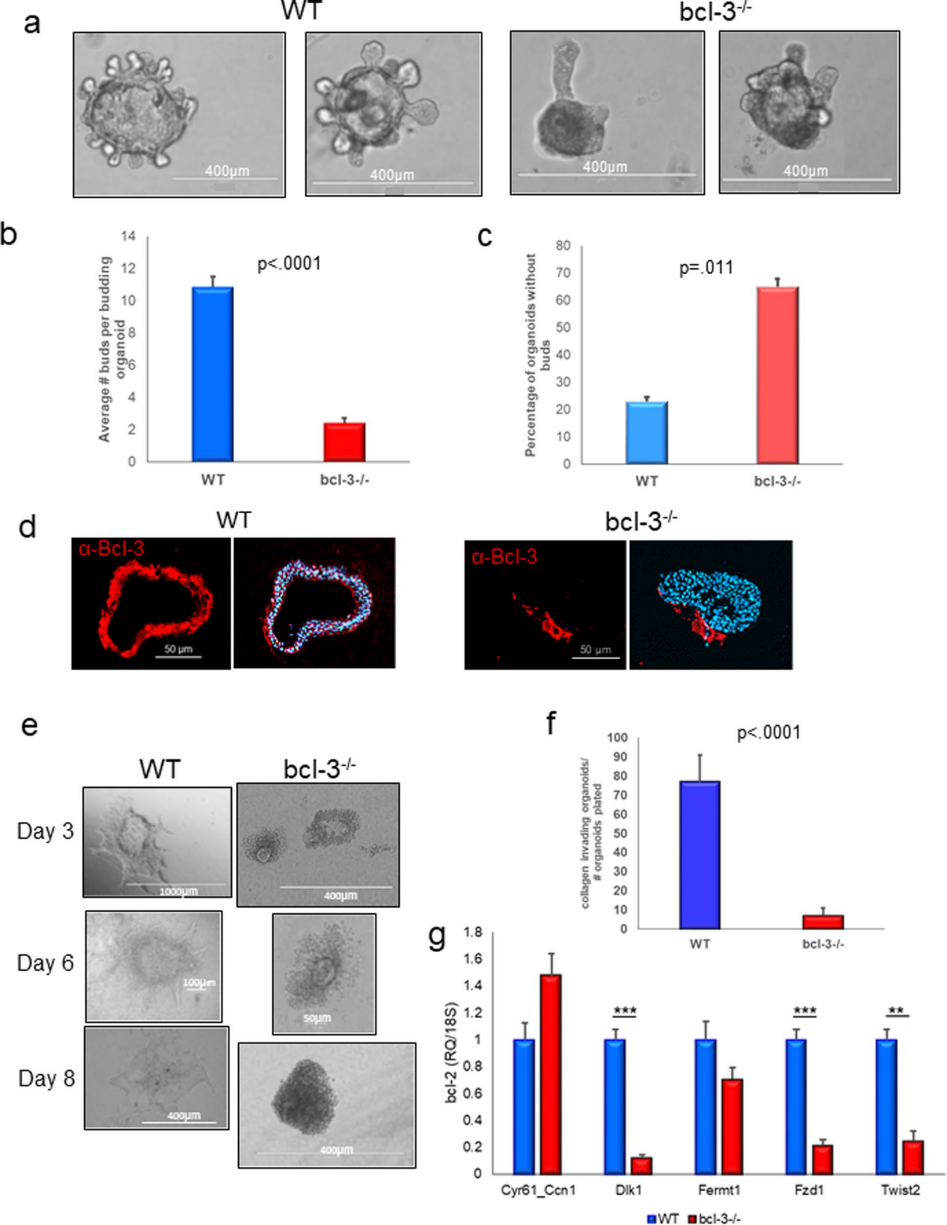
Bcl-3^−/−^ mammary epithelial organoids demonstrate retarded bud formation and defective collagen I invasion in 3D cultures. **a** *Bcl-3*^−/−^ and WT mammary organoids were isolated as described in Methods, seeded into Cultrex BME and allowed to grow for 10 days prior to imaging. Bars are 400 µm. **b** Graph depicting average number of organoid buds produced by mammary epithelium from WT and *bcl-3*^−/−^ mice after 10 days in basement membrane 3D cultures. Statistical analysis; unpaired *t* test. **c** Comparison of 10 day organoids from WT and *bcl-3*^−/−^ mice that lacked bud formation. Results of the paired *t* test, *n* = 3 mice per genotype. All bars are S.E.M. **d** Immunofluorescence for Bcl-3 on frozen sections of WT and *bcl-3*^−/−^ mammary organoids grown in Cultrex BME for 10 days and processed as described in Methods. DAPI was used to identify nuclei. **e** Mammary organoids were seeded into 3 mg/ml collagen I gels, and images were collected at the indicated days of culture. All images acquired with a 20× objective. **f** Graph showing number of organoids seeded relative to organoid numbers that produced continuous protrusive invasion at day 8 of culture. **g** qRT-PCR to detect the indicated invasion-associated genes in organoid RNA from 3 mice per genotype cultured for 8 days in collagen I. **p < 0.01; ***p < 0.001, unpaired *t* test

While in vivo analysis of mammary gland structure demonstrated defective ductal branching, at least some of this phenotype could be contributed by non-epithelial cells in the null animal. To assess cell autonomous ability of *bcl-3*^*−/−*^ mammary epithelium to undergo branching morphogenesis as compared to WT organoids, we cultured organoids in basement membrane hydrogels. Representative examples of branching organoids are shown in Fig. 2a. While WT organoids project multiple smaller buds, in some instances with secondary branches, *bcl-3*^−/−^ organoids were often devoid of buds and those organoids that did bud produced fewer and thickened primary structures only over the same period of time. Enumeration of organoid buds showed that the average number of primary and secondary mammary buds in WT cultures was significantly greater than *bcl-3*^*−/−*^ organoid cultures (Fig. 2b). Overall enumeration of organoids lacking bud formation showed that the majority (~ 65%) of  *bcl-3*^−/−^ organoids were devoid of buds compared to ~ 20% of WT organoids (Fig. 2c). Consistent for a role for Bcl-3 in bud formation, robust Bcl-3 immunofluorescence was obtained in frozen sections of WT organoids. Although some background staining was evident in acellular regions of the basement membrane hydrogel, as expected, no Bcl-3 was detected in cells within *bcl-3* null organoids (Fig. 2d). We also noted that *bcl-3*^*−/−*^ organoids demonstrated reduced luminal clearing, consistent with the relative lack of ductal branching structures. Overall, the reduced organoid budding in vitro is consistent with the attenuated mammary branching evident in the whole mount glands.

In the normal mammary gland, ducts transition from a polarized bilayer into a proliferative, motile, multilayered epithelium and then migrate collectively through the stromal tissue [42, 43]. Collagen I hydrogels mimic the stromal environment and induce collective invasion and dissemination of mammary epithelial organoid cells [44]. To test the cell autonomous ability of the mammary epithelial cells to invade extracellular collagenous stroma we cultured organoids from *bcl-3*^*−/−*^ [34] and WT mammary tissue in 3D fibrillary collagen I gels. At day 3 of culture, WT organoids produced protrusive extensions as previously described [44]; however, very few of the *bcl-3*^*−/−*^ organoids formed extensions and began to show evidence of collections of rounded-up, dissociated cells (Fig. 2e). By day 6, WT organoids formed large collective sheets of cells within the collagen matrix. However, *bcl-3*^*−/−*^ organoids failed to expand, demonstrating massive cell–cell dissociation. The percentage of organoids seeded that produced invasive fronts at day 6 of culture is depicted in the graph in Fig. 2f and demonstrates the marked inability of *bcl-3*^*−/−*^ mammary organoids to invade 3D collagen I. To assess whether dissociated cell accumulations in *bcl-3*^*−/−*^ collagen organoids were composed of significant numbers of dead or dying cells, we collected cells from gels following collagenase digestion and trypsin treatment and counted viable cells using trypan blue exclusion. Cells were > 95% viable, and no differences were seen between WT and *bcl-3*^*−/−*^ organoids (data not shown).

Wnt family signaling plays a major role in mammary side branching [45] as do members of the frizzled family of genes as well as twist and snail [46]. Evidence from public ChIP-seq datasets [47] suggests that Bcl-3 can bind directly to promoter regions of the same members of the frizzled gene family and the *twist2* and *snai1* genes as those described as up-regulated in terminal end buds (Table 1a). We selected several of these genes to assess their expression in collagen I organoid cultures. Previous reports indicated that Fzd1, Twist2 are both up-regulated in terminal end buds [46]. Twist family members promote EMT leading to cell migration and invasion [48], while Fzd1 is a canonical Wnt receptor that signals the transduction of downstream targets many of which are key elements in mammary epithelial cell migration and invasion [49]. qRT-PCR analysis in Fig. 2g shows that the relative expression of Fzd1 and Twist2 transcripts was significantly reduced in organoids lacking Bcl-3 compared to WT organoids. Previous reports showed that Dlk1 mRNA is reduced in collagen I relative to basement membrane organoid cultures, while Fermt1 and Cyr61 transcripts are normally increased (Table 1b). Interestingly, we found that Dlk1 (an inhibitor of Notch signaling) was significantly lower in *bcl-3*^*−/−*^ organoids relative to WT organoids, suggesting that Bcl-3 is likely to have a positive role in normal regulation of Dlk1. Fermt1 is involved in integrin signaling and epithelial–mesenchymal transition [50] and trended toward a reduced level in *bcl-3*^*−/−*^ mammary cells relative to WT cells but did not reach significance. Cyr61 mediates numerous cell functions including migration, adhesion and synthesis of the extracellular matrix through interactions with integrins and heparin sulfate proteoglycans [51], and expression was not significantly different from WT organoids in the absence of Bcl-3. Snai1 mRNA was undetectable in both sets of organoids (not shown).

**Table 1:** Bcl-3 gene targets identified under various experimental conditions. (a) Genes identified in Bcl-3 ChIP-seq datasets (Ref [47]) that correspond to; (a) genes up-regulated in terminal end bud/branching morphogenesis (Ref [46]); (b) genes differentially regulated in 3D collagen I cultures versus basement membrane (Ref [49]); (c) genes regulated after knockdown of Bcl-3 in breast cancer cells (Ref [53]). Genes in bold are up-regulated under the experimental conditions, and all others were down-regulated

(a) Mammary branching morphogenesis-associated gene expression. Up-regulated genes identified by both microarray of micro-dissected terminal end buds and Bcl-3 ChIP-seq data sets (Ref [43])	
**TWIST2**	Twist family bHLH transcription factor 2
**SNAI1**	Snail family zinc finger 1
**FZD1**	Frizzled class receptor 1
**FZD2**	Frizzled class receptor 2
(b) Collagen invasion-associated gene expression. Genes identified in both Bcl-3 ChIP-seq datasets and in microarray analysis of mammary organoids cultured in 3D ECM versus 3D collagen I (Ref [41])	
DLK1	Delta-like 1 homolog (Drosophila)
**FERMT1**	Fermitin family member 1
**CYR61**	Cysteine-rich, angiogenic inducer, 61
**PTGS2**	Prostaglandin-endoperoxide synthase 2 (prostaglandin G/H synthase and cyclooxygenase)
(c) Cell migration-associated gene expression. Genes identified in both Bcl-3 ChIP-seq datasets and in microarray analysis of gene expression following Bcl-3 knockdown in MDA231 (Ref [46]) and MG1361 breast cancer cells (Ref [32])	
**ARHGDIB**	Rho GDP dissociation inhibitor (GDI) beta
**RHOB**	Ras homolog family member B
**ARPC1A**	Actin-related protein 2/3 complex, subunit 1A, 41 kDa
**ARPC1B**	Actin-related protein 2/3 complex, subunit 1B, 41 kDa
**TUBB6**	Tubulin, beta 6 class V
**ARPC4**	Actin-related protein 2/3 complex, subunit 4, 20 kDa
**WASL**	Wiskott–Aldrich syndrome-like
**SSH2**	Slingshot protein phosphatase 2
**NME1**	NME/NM23 nucleoside diphosphate kinase 1
**NME2**	NME/NM23 nucleoside diphosphate kinase 2
**NME3**	NME/NM23 nucleoside diphosphate kinase 3
**ARHGDIB**	Rho GDP dissociation inhibitor (GDI) beta
CDC42	Cell division cycle 42
MYH9	Myosin, heavy chain 9, non-muscle
DIAPH1	Diaphanous-related formin 1
ENAH	Enabled homolog (Drosophila)
SSH3	Slingshot protein phosphatase 3
ARHGAP1	Rho GTPase activating protein 1
PAK1	p21 protein (Cdc42/Rac)-activated kinase 1
ARPC3	Actin-related protein 2/3 complex, subunit 3, 21 kDa
MYLK-AS1	MYLK antisense RNA 1
PFN2	Profilin 2

*Bcl-3*^*−/−*^ mammary cells were still able to detach from organoids without reduced viability but failed to migrate collectively into collagen. Several of the genes differentially regulated in mammary organoid collagen I cultures [52] are also detected by Bcl-3 ChIP-seq (Table 1b). Cell migration is largely mediated through Rho GTPase activity which is regulated by Cdc42 in a Bcl-3-dependent manner in breast cancer cell lines [53]. The same report identified several additional genes which displayed altered expression as a consequence of Bcl-3 knockdown, of which most are involved in the regulation of contractile proteins. A large number of these genes are present in the Bcl-3 ChIP-seq database [47] as putative direct targets of Bcl-3 (Table 1c).

Thus, Bcl-3 is involved in the regulation of several genes involved in both branching morphogenesis and collective and single cell migration into extracellular matrix.

### Virgin and pregnant *bcl-3*^*−/−*^ mammary glands demonstrate constitutively increased apoptosis and accelerated post-lactational involution

**Fig. 3:**
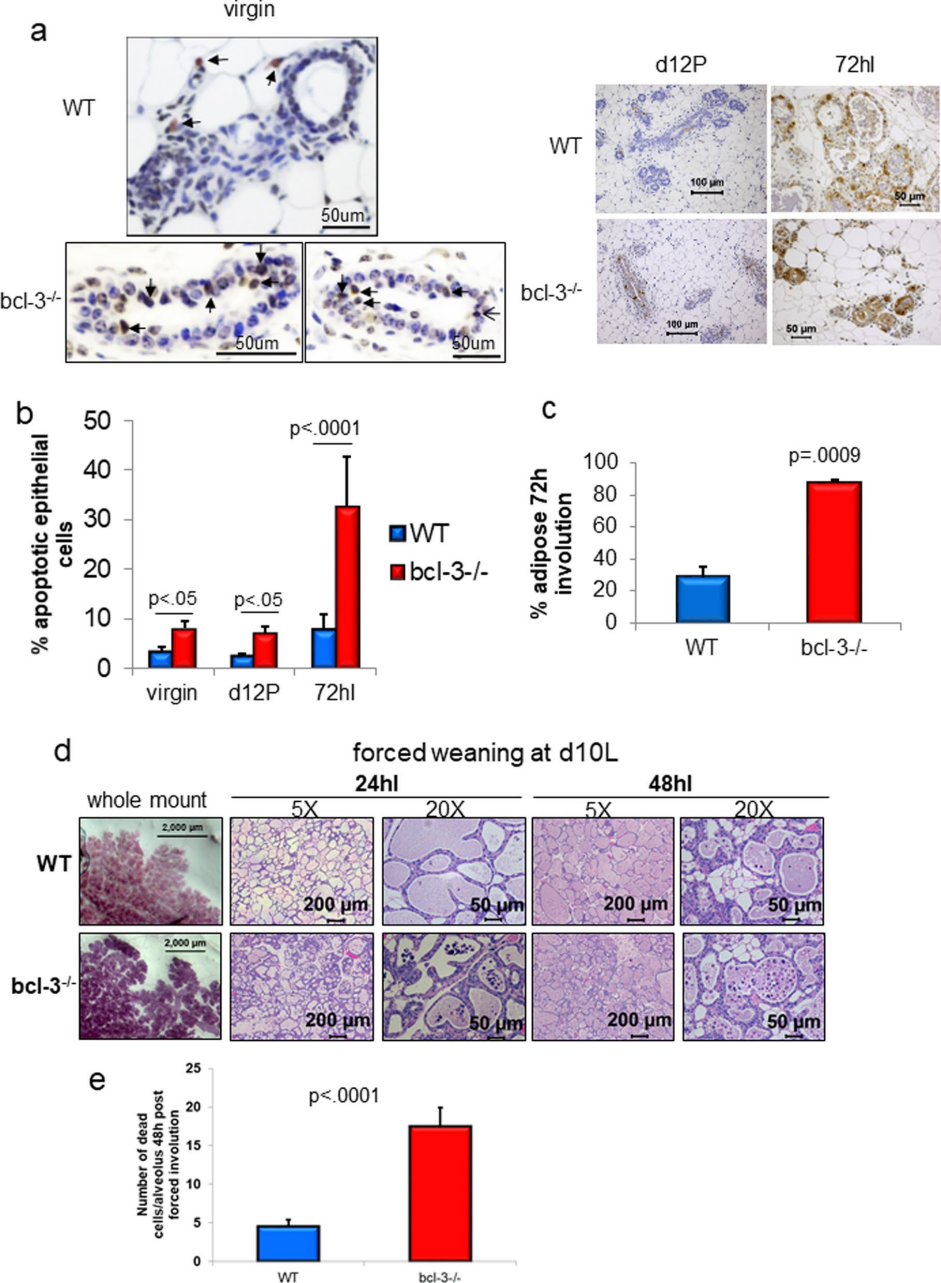
Virgin and pregnant *bcl-3*^*−/−*^ mammary glands show increased apoptosis and undergo accelerated involution. **a** Mammary gland sections were stained for apoptotic cells using Apoptag in virgin mice and at the indicated days of pregnancy, lactation and involution. **b** The percentage of apoptotic epithelial cells were enumerated from a minimum of 3 glands. Statistical analysis; one-way ANOVA. **c** The percentage of adipose tissue was determined in mammary gland sections at 72hI from 3 dams per genotype. Statistical analysis; unpaired t test. A minimum of 500 cells were enumerated per gland. **d** Pups were removed at d10L and mammary glands processed for whole mount at 24hI and H&E sections at 24hI and 48hI. Image magnification is shown above. **e** Mean numbers of dead cells in alveoli at 48 h post-forced weaning were determined from at least 50 alveoli per gland (*n* = 3). Statistical analysis; unpaired *t* test. All bars are S.E.M

In addition to defective invasive and branching properties of the *bcl-3*^*−/−*^ mammary epithelium, increased apoptosis may also be a contributing factor in the reduced complexity of *bcl-3*^*−/−*^ mammary glands. Assays for apoptotic cells in situ revealed significant levels of dead cells in *bcl-3*^*−/−*^ virgin glands and during pregnancy (d12P) (Fig. 3a, b). Apoptotic cells were not evident in WT or *bcl-3*^*−/−*^ lactating glands (d7.5L) which may reflect alternate pro-survival signaling in this phase or rapid removal by phagocytosis (not shown). Cell death in early involution occurs independent of classical apoptosis through a lysosomal pathway [54], while apoptosis is activated in the second phase [55]. Significantly increased numbers of apoptotic cells were clearly evident in *bcl-3*^*−/−*^ glands compared to WT at 72hI as a proportion of the remaining epithelial cells (Fig. 3a, b). In later stages of involution, adipose cells replace the milk-producing lobular structures [55] and by 72hI *bcl-3*^*−/−*^ mammary glands adiposity was already significantly increased compared to controls (Fig. 3c).

At 24 h post-weaning, cells in the normal mammary gland begin to detach with features typical of lysosomal cell death (swollen cells with two hypercondensed nuclei without membrane blebbing) [54]. Although early pre-weaning initiation of involution was apparent in *bcl-3*^*−/−*^ glands, we tested to see if the rate of involution was altered in these mice. We therefore performed forced weaning of pups at d10L. At 24 h, the involution in *bcl-3*^*−/−*^ glands was highly progressed with numerous detached cells showing binuclear condensation within alveoli, while in WT glands very few of these cells were evident. At 48hI, *bcl-3*^*−/−*^ mice continued to demonstrate significantly higher numbers of luminal dead cells compared with controls (Fig. 3d, e). Thus, Bcl-3 participates in the attenuation of cell death in phase I of involution.

### Bcl-3 regulates cell death signaling in the initial phase of involution

**Fig. 4:**
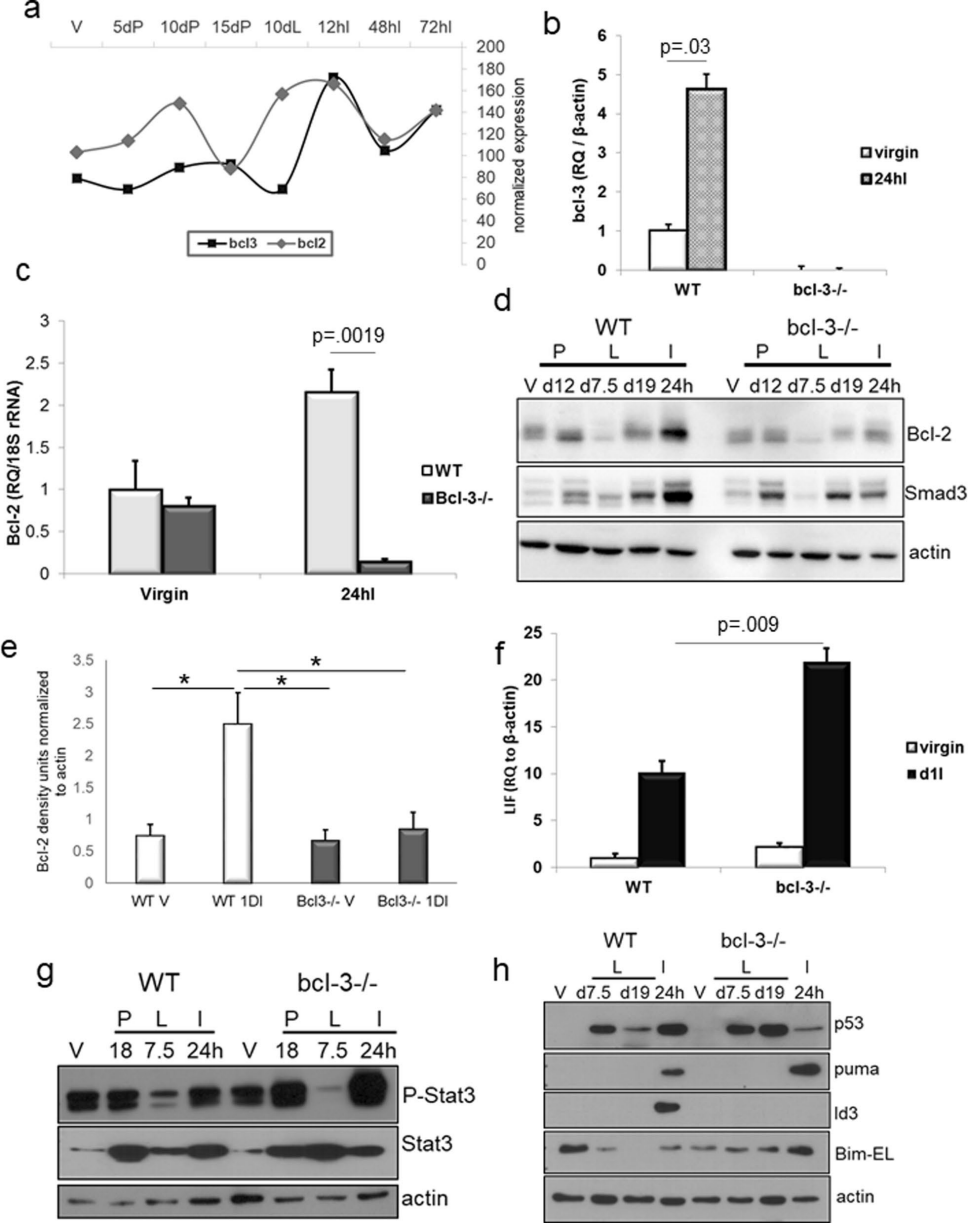
Absence of Bcl-3 accelerates and amplifies involution signaling events*.* **a** Graph of expression levels of mRNA for Bcl-3 and Bcl-2 in virgin mice and at the indicated times in pregnancy/lactation/involution cycle derived from public data sets [56]. (http://www.madgroup.path.cam.ac.uk/resources.shtml). **b** qRT-PCR analysis of Bcl-3 mRNA in virgin WT and *bcl-3*^*−/−*^ mammary glands and at 24hI (*n* = 3; statistical analysis, paired t test of the indicated means). **c** qRT-PCR for Bcl-2 in virgin WT and *bcl-3*^*−/−*^ mammary glands and at d19L and 24hI. Bars are S.E.M. of 3 mice performed in triplicate. Statistical analysis; one-way ANOVA. **d** Bcl-2 and Smad3 immunoblots were performed on 20 ug of whole gland lysate from virgin mice and at the indicated time points in WT and *bcl-3*^*−/−*^ mouse mammary glands. Blots are representative of results from 3 glands per genotype. **e** Densitometric analysis of Bcl-2 protein normalized to actin immunoreactivity from 3 independent experiments. Bars are S.E.M. *p < 0.05 One-way ANOVA. **f** LIF mRNA expression in WT and *bcl-3*^*−/−*^ virgin mice and at 24hI determined by qRT-PCR. Bars are S.E.M of 3 mice. Statistical analysis; paired t test of the indicated means. **g** Immunoblot of 20ug of whole gland lysate from WT and *bcl-3*^*−/−*^ mice for Stat3 and P-Stat3 at the indicated time points. Anti-actin reactivity was used as a loading control. **h** Immunoblots of p53, Id3, Puma and Bim-EL in virgin mice and at the indicated stages. For all immunoblots, actin was used as a protein loading control

An increase in Bcl-3 transcripts during the first phase of involution has been previously reported after forced weaning [16, 56]. The anti-apoptotic protein, Bcl-2, can be regulated by p52 homodimers in conjunction with its transactivation partner, Bcl-3, in both leukemic and breast cancer cells [17]. The graph in Fig. 4a of data from the Watson lab microarray dataset [16] indicates two peaks of Bcl-2 expression—one at day 10 gestation and the second higher peak coinciding with Bcl-3 expression at involution. We confirmed the increase in Bcl-3 mRNA at phase I involution in WT mice weaned at 21 days of lactation, whereas, as expected, no Bcl-3 transcript was obtained in *bcl-3*^*−/−*^ mouse mammary tissue (Fig. 4b). While Bcl-2 mRNA was increased in WT glands relative to virgin glands, at 24hI, *bcl-3*^*−/−*^ gland Bcl-2 transcripts did not show a similar increase (Fig. 4c). Consequently, relative to other stages of mammary lactational development, the induction of Bcl-2 protein was significantly greater in WT mammary tissue at 24hI than that achieved at 24hI in *bcl-3*^*−/−*^ glands where no increase relative to other stages was observed (Fig. 4d, e and Additional file 1: Fig. S2a). This data suggests that Bcl-2 is a key target for Bcl-3 expressed in early phase involution.

Bcl-3 has been shown to stabilize the transforming growth factor-β (TGFβ)-activated transcription factor, Smad3, in conjunction with metastatic spread of mouse mammary tumors [33]. While Smad3 protein levels were similar to control during pregnancy and at d19L when *bcl-3*^*−/−*^ mice are initiating involution, null mice failed to show the strong expression of protein at 24hI (Fig. 4d and Additional file 1: Fig. S2b). Although Smad3 contributes in a minor way to promote involution apoptosis [57], this result is consistent with a role for Bcl-3 in maintaining the stability of Smad3 protein during involution.

We also assessed additional markers of phase I involution. The secreted cytokine LIF is released in the first phase of post-lactational mammary regression and activates Stat3 phosphorylation promoting early lysosomal cell death [14]. qRT-PCR showed that LIF mRNA was significantly increased at 24hI in mammary glands from *bcl-3*^*−/−*^ mice relative to control (Fig. 4f). Consistent with this, P-Stat3 was markedly increased in *bcl-3*^*−/−*^ mice compared to controls at 24hI (Fig. 4g and Additional file 1: Fig. S2b).

p53 is a central regulator of cell death and is expressed during mid-lactation and in phase I of involution [58]. Although Bcl-3 can repress p53 through induction of MDM2 in various cancers as a consequence of DNA damage [19], immunoblot for p53 during lactation and after 24hI revealed similar expression in both mouse genotypes during lactation (Fig. 4h). In WT mice, p53 protein was only increased at 24hI coincident with the onset of involution. *Bcl-3*^*−/−*^ mammary glands exhibited an accelerated increase in expression at d19L which was strongly reduced by 24hI, consistent with the earlier onset of involution in mammary glands lacking Bcl-3. Bcl-3 has been shown to repress expression of the anoikis-inducing BH3-only protein Bim and Puma in T cells [59]. The BBC gene encoding the BH3-only protein, Puma, is a p53 regulated pro-apoptotic gene it is also identified as a Bcl-3 target by ChIP-seq [47]. Puma was increased at 24hI in both WT and *bcl-3* null mammary glands (Fig. 4h). Although the increase in Puma protein was consistently greater in 24hI *bcl-3*^*−/−*^ tissues compared to WT, this difference did not reach significance based on densitometric analysis across independent samples (Additional file 1: Fig. S2c). Pro-apoptotic Bim is necessary for mediating the formation of the ductal lumen by promoting detachment and anoikis-associated cell death in pubertal mice [60] and expression is a hallmark of anoikis [61]. Bim protein was differentially expressed at d19L in *bcl-3*^*−/−*^ glands but not WT glands and more highly expressed at 24hI compared to WT, consistent with the accelerated detachment of epithelium in knockout glands (Fig. 4h). Although the trend across independent samples for Bim proteins was toward increased expression at 24hI relative to other stages, whereas in WT mice it was reduced, based on immunoblot densitometry the difference between Bim protein level in WT and *bcl-3*^*−/−*^ 24hI glands did not quite reach significance (Additional file 1: Fig. S2c).

The inhibitor of differentiation protein 3 (Id3), is a target of Smad3 [62] and Id3 is present in the Bcl-3 ChIP-seq dataset [47]. We demonstrate here that Id3 was expressed in early phase involution coincident with Smad3 expression in WT mice but absent from *bcl-3*^*−/−*^ glands (Fig. 4h and Additional file 1: Fig. S2d). Interestingly, Id2 was shown to be important for cell survival during pregnancy [63]. Our result suggests that Id3 may have a similar role in the reversible phase of involution.

LIF is induced at weaning as a consequence of milk stasis-induced mechanical stretch in alveoli [64]. The evidence from H&E sections of lactating mammary glands shows that *bcl-3* knockout glands contain enlarged alveoli that are similar to those typical of early milk stasis. We speculate that the presence of already enlarged alveoli due to limited secondary branching results in profound induction of LIF in *bcl-3*^*−/−*^ mice as alveolar stretch at weaning is exacerbated. This results in strong activation of P-Stat3 accompanied by accelerated cell death in *bcl-3*^*−/−*^ mice. While the LIF gene does not appear in either the Bcl-3 ChIP dataset, nor does it have regulatory elements that correspond to NF-κB binding sites, we cannot formally rule out that enhanced LIF induction is not downstream of Bcl-3 regulated signaling.

Overall, our results are consistent with Bcl-3 regulation of cell survival through induction of Bcl-2 and Id3 proteins (the latter through transcriptional means or indirectly through Smad protein stabilization) combined with attenuated Puma and Bim expression to protect cells from wide-scale apoptotic cell death in the first stage of involution.

### Increased ratio of mammary squamous to adenocarcinoma lesions in DMBA-treated *bcl-3*^*−/−*^ mice

As described, Bcl-3 can be detected in human breast cancers and breast cancer cell line xenograft tumors [35, 65]. To assess the role of Bcl-3 in tumorigenesis, we utilized the MPA/DMBA carcinogen-induced mouse mammary tumor model to generate stochastic tumors. Following the final treatment, the latency to detection of a palpable lesion was variable but not significantly different between WT and *bcl-3*^*−/−*^ mice (mean 100.8 days; 1.7 tumors per *bcl-3*^*−/−*^ mouse vs. mean 99.8 days; 1.4 tumors per WT mouse- data not shown).

**Fig. 5:**
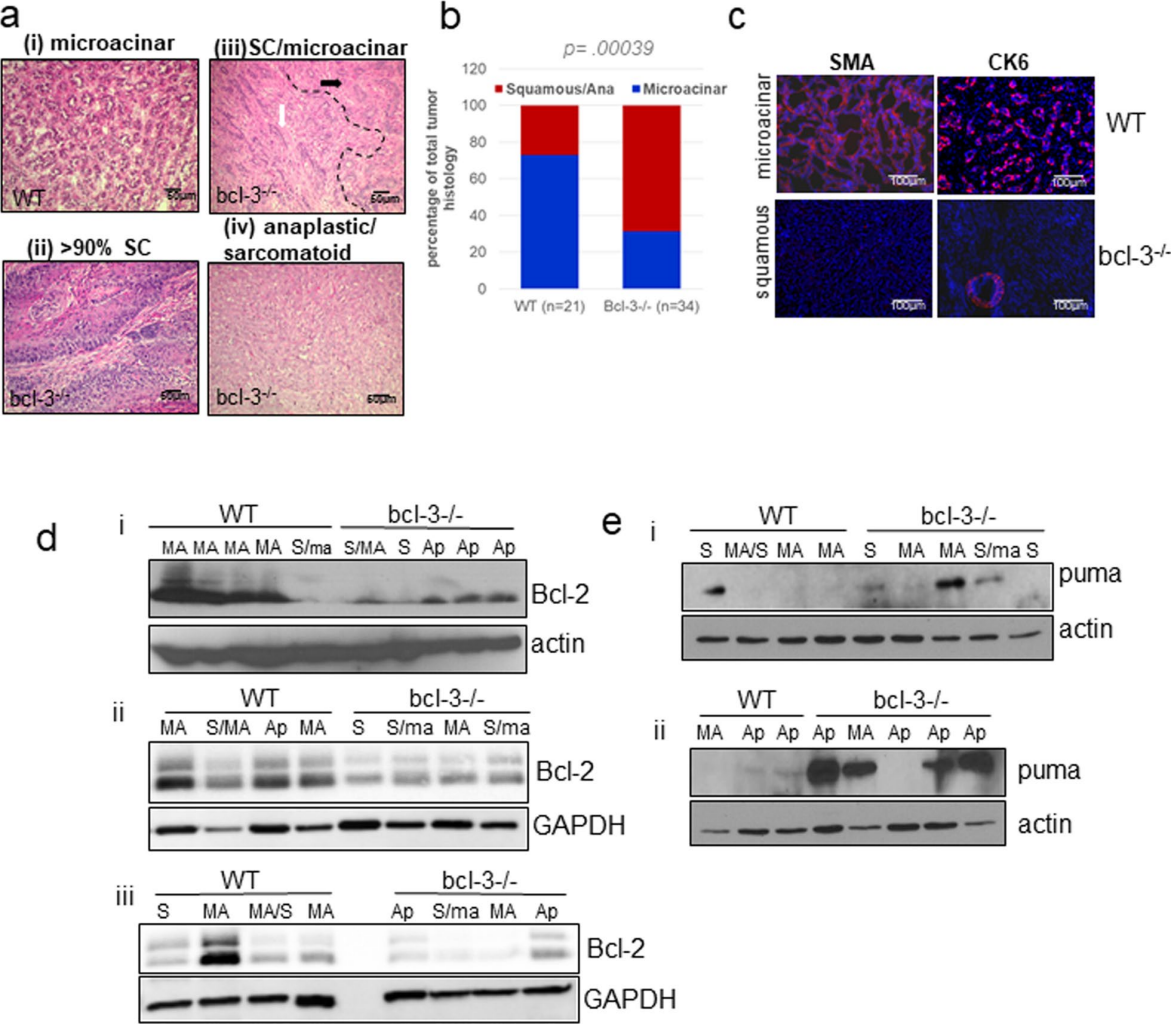
*Bcl-3* null mice preferentially develop a higher percentage of carcinogen-induced mammary squamous carcinomas lacking NF-κB activity. **a** Representative H&E-stained paraffin sections of mammary tumors from WT and *bcl-3*^*−/−*^ mice showing (i) adenocarcinoma (microacinar pattern); (ii) predominantly squamous carcinoma; (iii) squamous (white arrow) mixed with microacinar (black arrow) adenocarcinoma; and (iv) anaplastic tumor (sarcomatoid pattern). Bar = 50µ. **b** Histogram depicting the percentages of total squamous/anaplastic versus microacinar adenocarcinoma histology between WT and *bcl-3*^*−/−*^ tumors. Significantly more squamous histology was present in *bcl-3*^*−/−*^ mammary tumors, statistical analysis; unpaired *t* test. **c** Immunofluorescence staining for smooth muscle actin (SMA) and cytokeratin-6 (CK6) in representative cryosections of microacinar versus squamous tumors. Note SMA positive cells in a ductal structure within the squamous tumor section. Bars = 100µ. **d** Immunoblots for Bcl-2 in the indicated lesions from *bcl-3*^*−/−*^ and WT mice. Note the low levels of Bcl-2 in anaplastic and squamous tumors relative to microacinar tumors. **e** Anti-puma immunoblots of *bcl-3*^*−/−*^ and WT in the indicated lesions (panels i and ii). Lowercase histology letters in mixed tumors indicate a less than 30% proportion of the tumor composition

Tumor histopathologies were initially reviewed on hematoxylin and eosin-stained sections (Fig. 5a). The most prominent phenotype produced in control mice was a microacinar (MA) basaloid adenocarcinoma consisting of acini of single layers of epithelial cells (panel i). In contrast, although MA lesions were also present in some *bcl-3*^*−/−*^ mice, tumors were composed of a large proportion of poor to well-differentiated squamous (S) cell carcinomas (panel ii). More than 75 percent of *bcl-3*^*−/−*^ tumors had a significant (> 30% of the lesion) squamous cell component mixed with the MA pattern (panel iii). In addition, nearly 25 percent of *bcl-3*^*−/−*^ tumors were anaplastic (Ap) with a sarcomatoid (spindle) pattern (panel iv) compared to less than 5 percent of the control tumors. The relative levels of MA and S/Ap histologies across tumors in WT and *bcl-3*^*−/−*^ mice are shown in Fig. 5b. Tumors were further characterized by immunohistochemistry. Adenocarcinoma MA lesions immunostained positive for α-smooth muscle actin (SMA) and basal cytokeratin-6 (CK6) in large focal regions, consistent with the prominent myoepithelioid and basal epithelial components [66]. In contrast, regions encompassing squamous cell carcinomas were negative for both SMA and CK6 (Fig. 5c).

Mammary squamous carcinomas are classified as metaplastic and are thought to derive from the stem/basal cell mammary population, whereas basal adenocarcinomas are derived from a luminal progenitor subset [20, 67]. Thus, the enhanced proportion of squamous carcinomas in *bcl-3*^*−/−*^ mice might have been due to increased numbers of cells of origin. We therefore assessed the expression of Bcl-3 in different mammary progenitor populations using public gene expression array data for human [68] and mouse [69] mammary epithelial cell subpopulations. Indeed, Additional file 1: Fig. S3a shows that BCL-3/Bcl-3 expression progressively increases from lowest levels in mammary stem cell (MSC)-enriched populations to highest levels in mature luminal cells in both human and mouse glands. Thus, Bcl-3 appeared to be differentially associated with maturation of luminal progenitors. However, FACS analysis to detect populations enriched for MaSC, LP and mature luminal cells (ML) showed no difference between the ratios of MaSC to LP/ML in *bcl-3*^*−/−*^ glands relative to control glands (Additional file 1: Fig. S3b, c). Thus, the absence of Bcl-3 did not impact the relative differentiation of mouse luminal mammary epithelial cells.

To assess whether the absence of Bcl-3 altered Bcl-2 expression in mammary tumors as it did during involution, we performed immunoblot for Bcl-2 in different tumor subtypes from *bcl-3*^*−/−*^ and control mice. Figure 5d shows that MA and squamous tumors from control animals express higher levels of Bcl-2 compared to all tumor subtypes from *bcl-3*^*−/−*^ animals. Interestingly, Bcl-2 expression was reduced in the pure squamous or mixed tumor tissue from WT mice relative to MA, consistent with a lack of requirement for Bcl-2 in the squamous lesions. Overall these results support a role for Bcl-3 in the regulation of non-canonical and canonical NF-κB activity and Bcl-2 expression in mammary adenocarcinomas such that its absence results in preferential expansion of squamous tumors lacking both NF-κB activity and Bcl-2 expression.

Since Puma was increased in *bcl-3*^*−/−*^ during mammary involution relative to controls, we also assessed Puma expression in tumors (Fig. 5e). Puma was detected in approximately 70% of *bcl-3*^*−/−*^ tumors independent of histotype, and expression was associated with strongly increased p53 expression in only one sample from each genotype.

Although Bcl-3 can inhibit expression of p53 protein in response to DNA damage [19], the presence of p53 in tumors did not correlate with the *bcl-3* genotype in mammary tumors (Additional file 1: Fig. S4)*.* Instead expression was more strongly correlated with tumor phenotype, wherein p53 protein was very low to absent from squamous tumors from either mouse. The anaplastic tumors expressed a spectrum of p53 levels, likely a function of p53 mutations. Interestingly, similar to involuting glands, a subset of adenocarcinomas and anaplastic tumors in *bcl-3*^*−/−*^ mice expressed elevated levels of Puma compared to WT tumors. Given the reduced Bcl-2 protein and increased expression of Puma in *bcl-3*^*−/−*^ tumors, Bcl-3 likely contributes to tumor cell survival, in part, through the activation and repression of these genes, respectively.

**Fig. 6:**
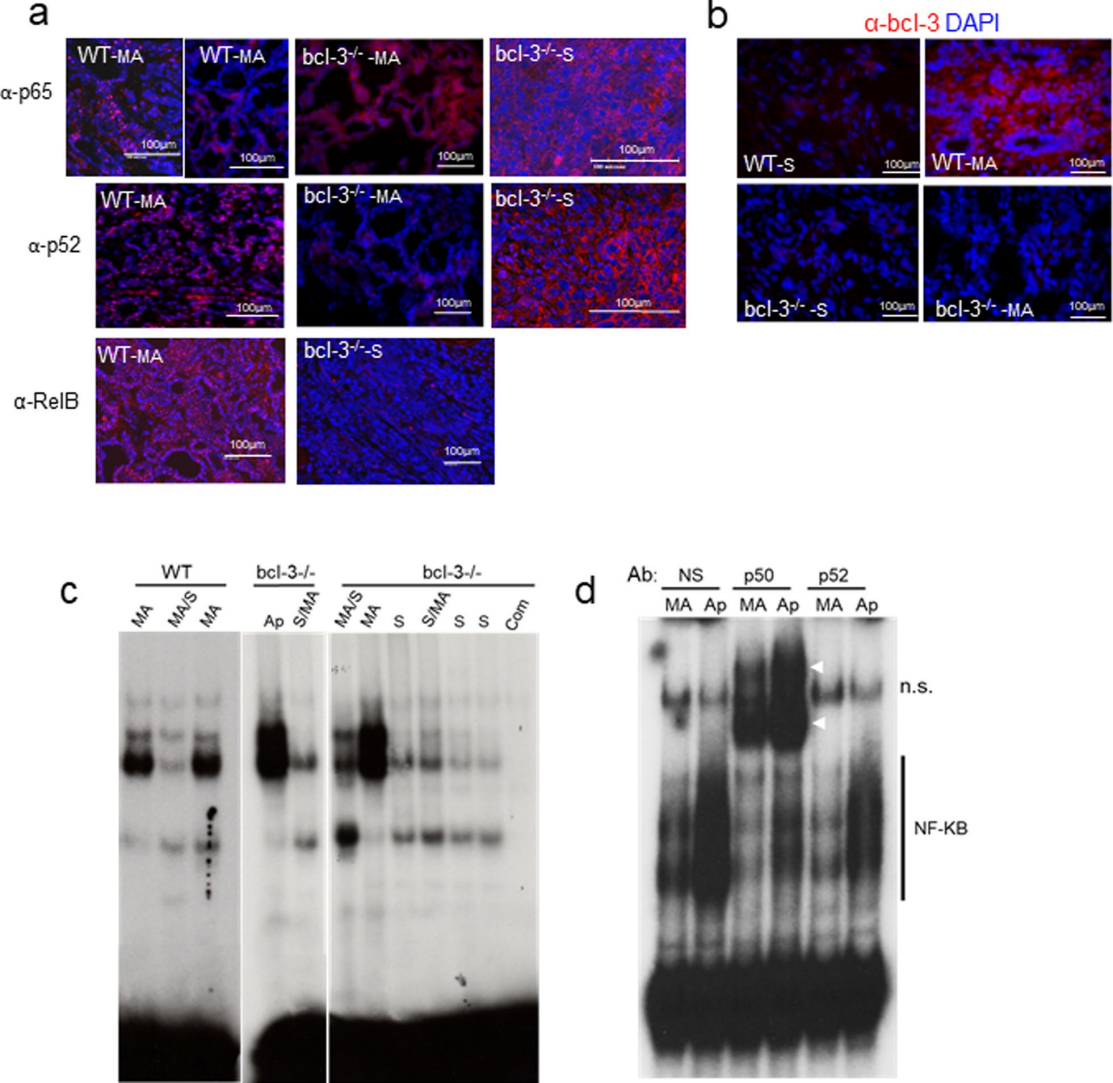
NF-κB is activated in mammary adenocarcinomas but not squamous cancers. **a** p65, p52 and RelB NF-κB immunofluorescence in microacinar (MA) and squamous (S) tumors from the indicated mice. Note the strong nuclear staining for p52 in WT MA versus *bcl-3*^*−/−*^ MA, the presence of cytoplasmic p65 and p52 and absence of RelB in squamous tumors. **b** Anti-Bcl-3 immunofluorescence in S and MA tumors from WT and *bcl-3*^*−/−*^ mice. All bars = 100µ. Pixels from expanded boxed areas show overlap between chromatin (DAPI) and anti-Bcl-3 IF within WT MA tumor cells positive for Bcl-3. **c** EMSA analysis of nuclear extracts from the indicated tumor subtypes. S/MA > 70% squamous; MA/S > 70% microacinar; Ap, anaplastic. The NF-κB consensus sequence was used as a DNA probe. Com: cold probe was incubated with labeled probe and WT (MA tumor) nuclear extract prior to gel loading. **d** Supershift analysis using normal serum (NS), anti-p50 or anti-p52 incubated with nuclear extracts from MA and Ap tumors and labeled NF-κB probe. NF-κB complexes are indicated. White arrows indicate supershifted complexes. n.s. non-specific bound probe

We next investigated expression of NF-κB proteins using immunofluorescence to detect NF-κB proteins p65/RelA, NF-κB2/p52 in tumors from MA and squamous tumors. The results in Fig. 6a show that robust staining of p52 and RelB is nuclear in the MA tumors in both the cells surrounding the glandular structures as well as the parenchymal regions when present on the slide. Nuclear p65/RelA canonical NF-κB was also detected in both WT and *bcl-3*^*−/−*^ tumors. The squamous tumors, found predominantly in *bcl-3*^*−/−*^ mice, expressed both p52 and p65; however, both proteins were excluded from the nucleus. Squamous tumors also did not express RelB. Immunostaining for Bcl-3 showed nuclear staining in many cells within WT MA lesions but not in squamous lesions or, as expected, not in *bcl-3*^*−/−*^ tumors (Fig. 6b). EMSA of tumor nuclear extracts using a canonical NF-κB response element confirmed the presence of activated NF-κB in MA but not squamous tumors (Fig. 6c). Anaplastic tumors frequently contained high levels of nuclear NF-κB regardless of *bcl-3* genotype. The probe shift mediated by NF-κB was verified by incubating complexes with anti-p50 and anti-p52. Anti-p50 supershifted much of the complexes in these samples, while anti-p52 disrupted a portion of the complexes (Fig. 6d).

## Discussion

The studies reported here reveal the multi-functional roles of Bcl-3 in mammary gland biology and tumorigenesis. The most striking defect in *bcl-3*^*−/−*^ mammary epithelium is essentially the inability of these cells invade 3D collagen I gels in vitro. In contrast to WT organoids, *bcl-3* null organoids demonstrated an intrinsic inability to form protrusive invasive extensions into fibrillar collagen. The vast majority of *bcl-3*^*−/−*^ organoids resolved into rounded-up localized accumulations of detached cells after 24 h of culture. Moreover, these organoids are also deficient in primary and secondary budding into basement membrane gels, a feature that was recapitulated in the reduced branching we observed in *bcl-3*^*−/−*^ mammary glands. In the mature virgin and pregnant *bcl-3*^*−/−*^ mammary glands, significantly higher numbers of apoptotic cells were detected compared to WT glands consistent with a constitutive pro-survival function of Bcl-3. Thus, deficiency in branching morphogenesis, ECM/stromal invasion and enhanced cell death collectively could contribute to the reduced complexity in *bcl-3*^*−/−*^ mammary glands.

During breast cancer pulmonary metastasis in mice, TGFβ signaling stabilizes Smad3 necessary for metastasis in a Bcl-3-dependent manner [33]. While Smad3 has a minor role in apoptotic cell death during involution [57], we noted a profound decrease in Smad3 at 24hI in *bcl-3*^*−/−*^ mammary glands relative to WT mice, consistent with a similar role for Bcl-3 in the regulation of Smad3 in the normal mammary gland. The Id proteins are also regulated through TGF-β signaling and similar to observations in the metastatic tumor model where Id3 was reduced in *bcl-3*^*−/−*^ mammary tumors, *bcl-3* null mammary glands were devoid of Id3 expression at involution, while expression was strongly increased in WT glands. Importantly, TGF-β and Smads also have key roles in mammary branching morphogenesis [70]. Thus, our data suggest that Smad3 signaling during involution, and ostensibly during branching morphogenesis, IS at least partially dependent on the presence of Bcl-3.

Bcl-3 protein is nearly undetectable in normal cells and is rapidly degraded via the ubiquitin–proteasome system triggered as a consequence of GSK3-mediated phosphorylation of Bcl-3 [71]. As discussed, Wnt signaling is also critical for mammary side branching [45] and suggests that Bcl-3 protein is thus stabilized during branching morphogenesis and able to participate in the transduction of *twist1* and *snai1* and frizzled family genes that have roles in this process [71]. Our finding that the absence of Bcl-3 correlates with a decrease in both *twist1* and *fzd1* gene expression relative to WT mammary cells upon exposure to collagen I suggests that the defective invasion phenotype is associated with reduced expression of these important migration-associated genes. During phase II, involution proceeds with massive death of the secretory epithelium and dramatic tissue remodeling as elevated levels of fibrillar collagen are laid down by fibroblasts immediately adjacent to the collapsing alveoli [72]. Involution stroma has been shown to promote the invasion of tumor cells resulting in metastatic disease [72], a function that is severely attenuated in *bcl-3* null mouse mammary tumor cells [32, 33]. Pups grew normally in *bcl-3*^*−/−*^ litters; however, the reduced branching complexity in the *bcl-3*^*−/−*^ mammary glands appears to have resulted in numerous hyper-enlarged alveoli. The engorgement in the last week of lactation precipitating robust induction of LIF/Stat3 is a phenotype similar to that we described previously in mice null for the anti-apoptotic protein, cIAP2 [73].

The Id proteins are also implicated in cell survival and Bcl-3 can directly bind the Id1, Id2 and Id3 promoters [47, 74]. The data provided here demonstrating that Id3 is induced in phase I involution suggest that it may have a similar survival role during this reversible phase. Apoptosis subsequent to milk engorgement is also associated with the induction of Bim [75] which, along with Puma, trended toward increased expression at 24hI in *bcl-3*^*−/−*^ glands but not WT glands compared to other stages of development. Bcl-2 gene expression is induced within 12 h of weaning (Fig. 4b and [16]) and overlaps at both the mRNA and protein levels (shown here) with that of Bcl-3. The NF-κB binding site of the Bcl-2 promoter is specifically bound and transactivated by p50 or p52 homodimers through association with Bcl-3 [17]. Consistent with this, our data support the contention that *bcl-2* is an in vivo target gene for p105/p50 in the first 24 h post-weaning where a p50-Bcl-3-Bcl-2 axis would contribute to cell survival during the initial, reversible, phase of involution. Thus, the rapid onset of apoptosis in *bcl-3*^*−/−*^ glands may be a consequence of both the extent of engorgement within the enlarged alveoli coupled to a deficit in NF-κB/Bcl-3-dependent up-regulation of Bcl-2 and Id3 coupled to a reduction in Bim and Puma gene repression. The combination of all factors including reduced survival and enhanced pro-apoptotic factors could explain the rapid and wide-spread cell death within 24 h of weaning in *bcl-3* null mice. Although the induction of canonical NF-κB (NFKB1) is critical for cell death in the early stages of involution [38], Bcl-3 appears to mitigate this signaling to prevent rapid cell death.

Bcl-3 is expressed in human breast cancers [35, 65] and is associated with poor prognosis [33]. We found that absence of Bcl-3 results in tumors with higher proportions of squamous carcinoma or anaplastic sarcomatoid lesions relative to the microacinar basaloid adenocarcinomas. We showed that squamous tumors are devoid of nuclear NF-κB activity, whereas, consistent with our previous findings [76], the basal adenocarcinomas expressed high levels of nuclear alternative (p52/RelB) NF-κB. Thus, the high proportions of *bcl-3*^*−/−*^ squamous lesions may reflect a requirement for Bcl-3 for optimal NF-κB signaling to support adenocarcinoma cell survival and tumor progression.

## Conclusions

In summary, these studies demonstrate that Bcl-3 has multiple functions in the normal mammary gland where it participates in side branching in the virgin and pregnant mammary gland and also performs a critical role in stromal matrix invasion. The latter confirms that the role of Bcl-3 in collagen invasion is fundamental not only to tumor cells but also normal mammary epithelial cells. Further, Bcl-3 promotes cell survival during involution where it retards apoptosis in phase I of involution at least in part through induction of Bcl-2 and Id3 and repression of Bim and Puma. Further studies should test the ability of *bcl-3*^*−/−*^ dames to reinstate lactation upon reintroduction of pups 24–36 h following the onset of involution. Lastly, stochastic tumor formation in *bcl-3* null and WT mice where there is no apparent difference in the relative proportions of progenitors as cells of origin for tumors suggests that Bcl-3 could be a key accessory factor in pro-survival and epithelial gene expression that ultimately determines the landscape of mammary tumor pathology. Bcl-3 clearly has a role in regulating invasion and has been proposed as a target for prevention of tumor metastasis. While we cannot rule out the possibility that the propensity toward squamous tumors in *bcl-3*^*−/−*^ mice is due to overgrowth of less viable epithelial tumors, it is possible that strategies to reduce Bcl-3 activity may paradoxically select for a mesenchymal tumor phenotype. To this end, it would informative to isolate *bcl-3* null squamous mammary tumor cells and determine whether ectopic Bcl-3 expression has a direct role in reversibility of the mesenchymal cancer cell phenotype by activating genes such as *Fzd1* and *Twist2*.

## Supplementary Information

**Additional file 1. Figure S1:** Estrus staging of mice for whole mount analysis. **Figure S2.** Biological repeat immunoblot experiments. **Figure S3.** Bcl-3 expression in human and mouse mammary progenitors and mature cells and population analysis. **Figure S4.** Immunoblot for p53 in mammary lesions from WT and *bcl-3*^−/−^ mice.

## Data Availability

Public datasets used to identify Bcl-3 ChIP-seq targets are available at: https://maayanlab.cloud/Harmonizome/gene_set/BCL3/ENCODE+Transcription+Factor+Targets.

## Abbreviations

WT: Wild-type control mouse
ChIP-seq: Chromatin immunoprecipitation-sequenced
LIF: Leukemia inhibitor factor
SMA: α-Smooth muscle actin
CK6: Cytokeratin-6
NF-κB: Nuclear factor kappa B
Stat3: Signal transducer and activator of transcription 3
Smad3: Mothers against decapentaplegic homolog 3
TGFβ: Transforming growth factor-β
Id3: Inhibitor of differentiation 3
MA: Microacinar adenocarcinoma
S: Squamous carcinoma
Ap: Anaplastic carcinoma
