# Supplementary material for: Multiple roles for Bcl-3 in mammary gland branching, stromal collagen invasion, involution and tumor pathology
Source: Breast Cancer Res. 2022 Jun 9;24:40. doi: 10.1186/s13058-022-01536-w (PMC9185916; doi:10.1186/s13058-022-01536-w)
Supplement: Supplementary file 1 — Additional file 1. Figure S1: Estrus staging of mice for whole mount analysis. Figure S2. Biological repeat immunoblot experiments. Figure S3. Bcl-3 expression in human and mouse mammary progenitors and mature cells and population analysis. Figure S4. Immunoblot for p53 in mammary lesions from WT and bcl-3−/− mice. [file 13058_2022_1536_MOESM1_ESM.pdf]

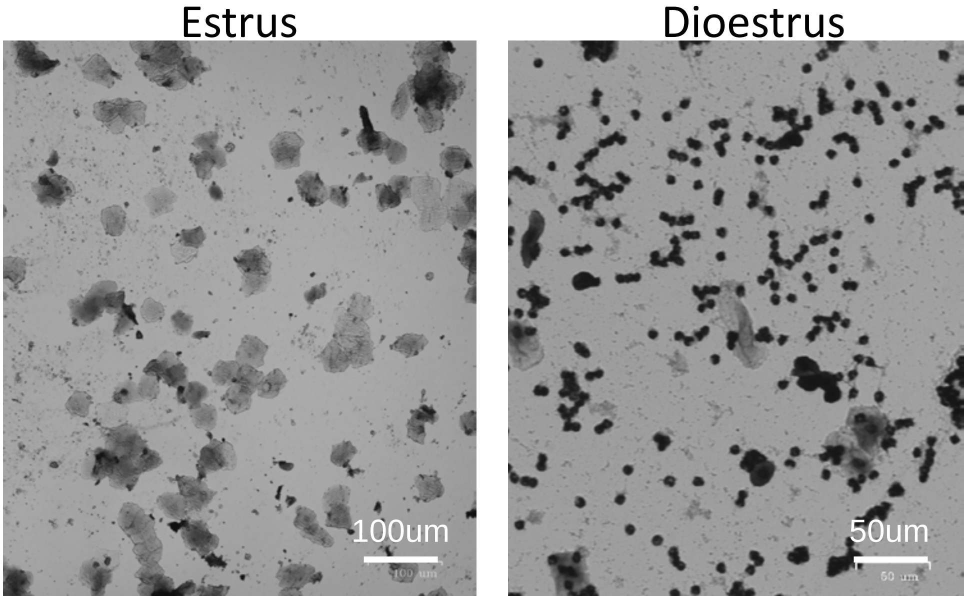

Figure S1: Estrus staging of mice for whole mount analysis: Vaginal washes were performed and cells on coverslips were stained to determine estrus cycle stage. Estrus and dioestrus are shown.

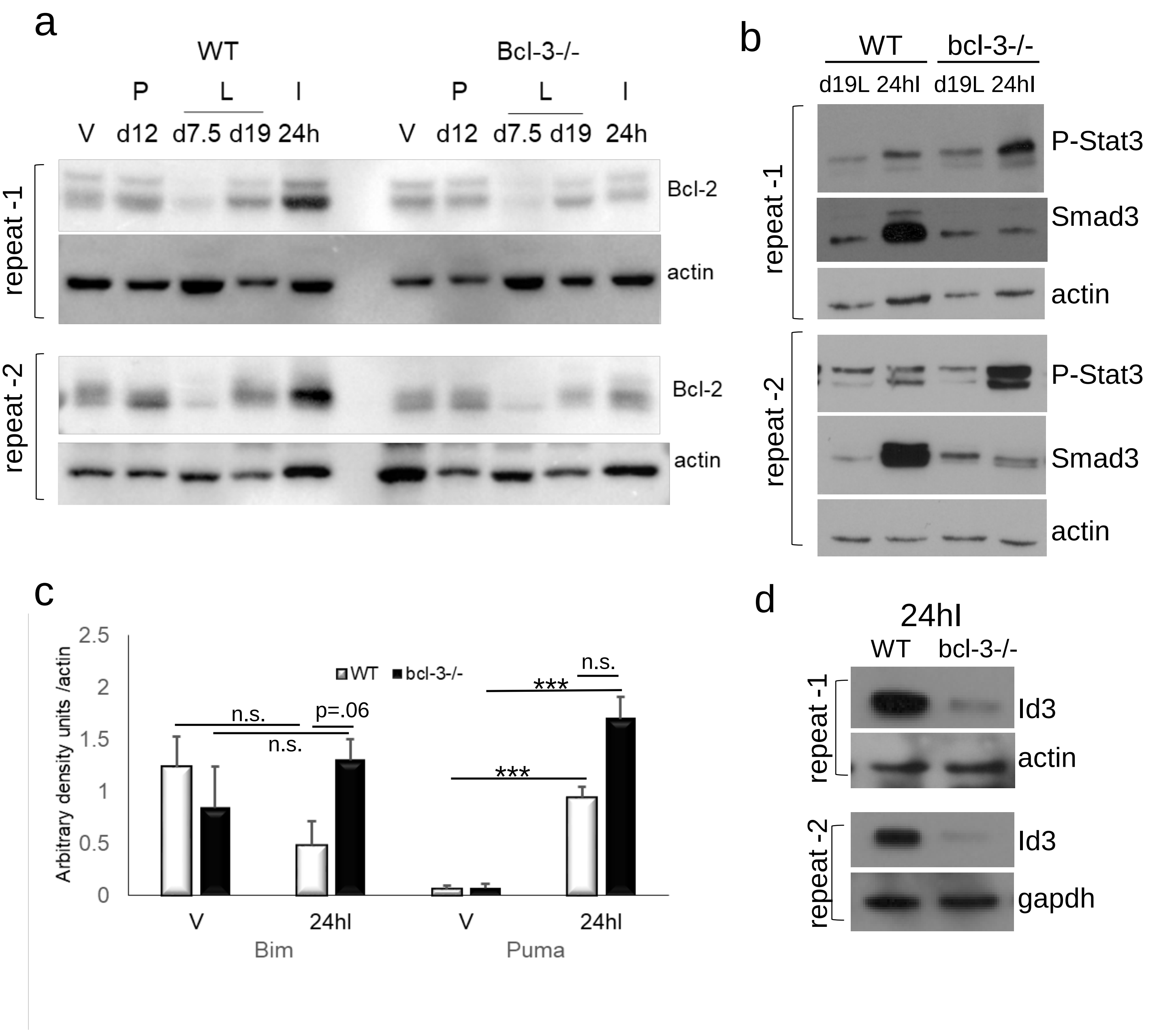

Figure S2. Biological repeat immunoblot experiments. 20ug each of WT and *bcl-3*$^{-/-}$ mammary gland lysates at d19L and 24hI were subjected to immunoblot for: **a**, Bcl-2; **b**, P-Stat3, Smad3. **c,** Densitometric analysis of Bim and Puma protein immunoblots from virgin and 24hI in WT and *bcl-3*$^{-/-}$ mice. (n.s.- not significant) One-way ANOVA. **d,** Id3 immunoblot from mammary gland lysates at 24hI. Actin or gapdh were used as loading controls as indicated.

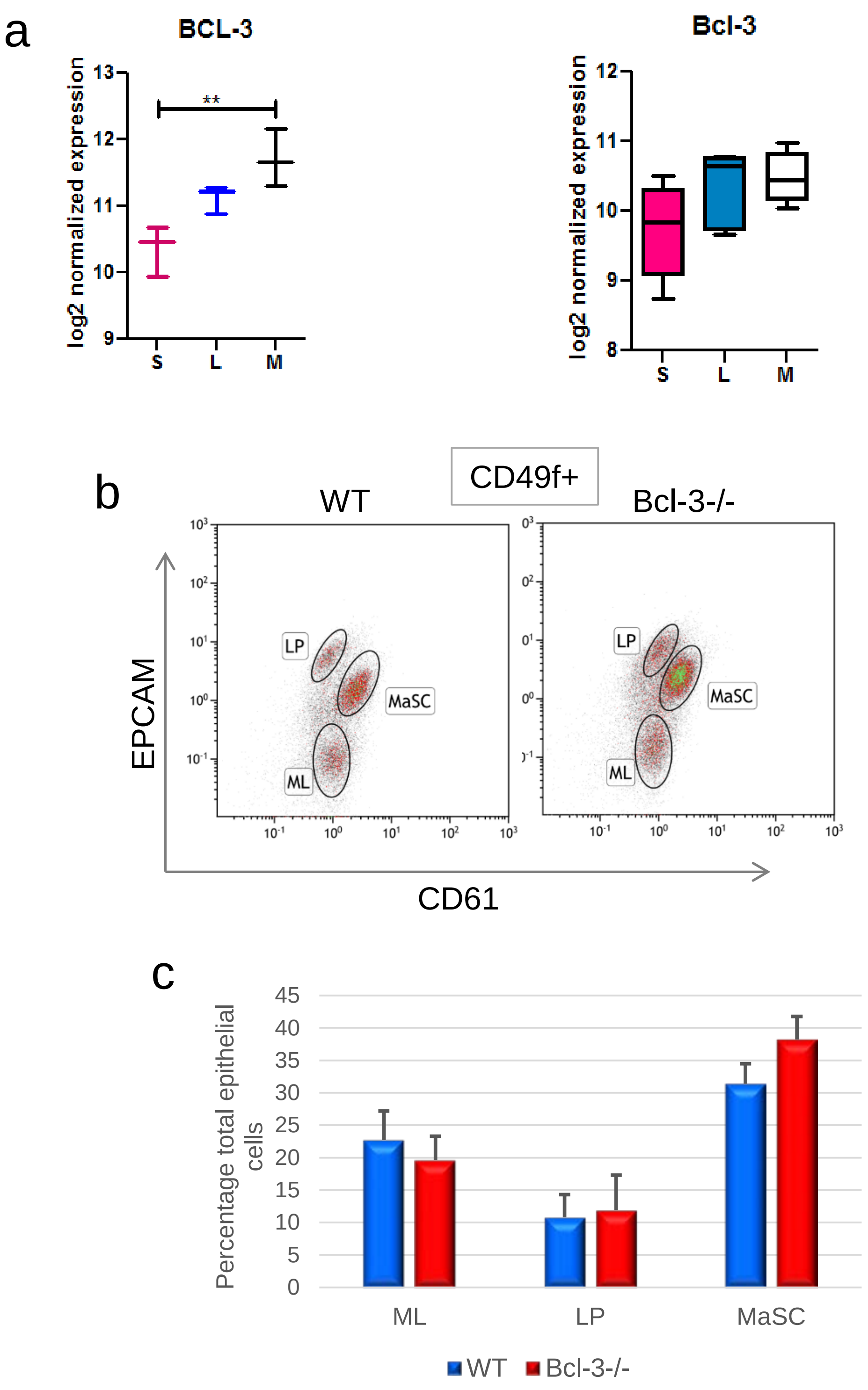

Figure S3. Bcl-3 expression in human and mouse mammary progenitors and mature cells and population analysis. A, Published microarray data for human [Ref 68] and mouse [Ref 69] enriched mammary epithelial cell subpopulations (S, stem cell/basal progenitors; L, luminal progenitors; M, mature cells) was analyzed and box plots of gene expression of human BCL-3 and /mouse Bcl-3 were generated. (**$p<.01$, paired t-test). B, FACS analysis of epithelial populations in WT and Bcl-3-/- mouse mammary glands. MaSC, mammary stem cells; LP, luminal progenitors; ML, mature luminal cells. C, Histogram depicting percentage of cells in FACS subpopulations (n=3). No significant difference was determined.

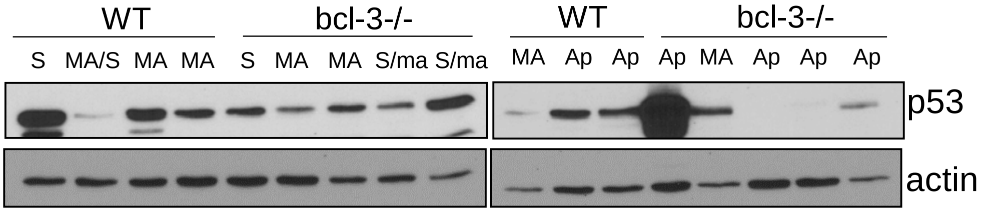

Figure S4. Immunoblot for p53 in mammary lesions from WT and bcl-3$^{-/-}$ mice. 20ug of whole tumour lysate was subjected to immunoblot for p53. Lower case letters indicate a smaller proportion of histology within the tumor composition.
